# A novel algorithm for high compression rates focalized on electrical power quality signals

**DOI:** 10.1016/j.heliyon.2021.e06475

**Published:** 2021-03-12

**Authors:** Milton Ruiz, Silvio Simani, Esteban Inga, Manuel Jaramillo

**Affiliations:** a Universidad Politécnica Salesiana, Quito, Ecuador; b University of Ferrara, Ferrara, Italy

**Keywords:** Compression algorithms, Data compression, Data processing, Power quality (PQ), Wavelet transforms

## Abstract

This research proposes a high-performance algorithm for the compression rate of electrical power quality signals, using wavelet transformation. To manage the massive amount of data the telecommunications networks are constantly acquiring it is necessary to study techniques for data compression, which will save bandwidth and reduce costs extensively by avoiding having massive data storage facilities. First biorthogonal wavelet level six transform is applied, however after compression, the reconstructed signal will have a different amplitude and it will be shifted when compared to the original one. Then, normalization is used (for amplitude correction between the original signal and reconstructed one) by multiplying the reconstructed signal by the result of the division between the original signal maximum magnitude and the reconstructed signal maximum magnitude. Thirdly, the ripple in the reconstructed signal is eliminated by applying a moving average filter. Finally, the shifting is corrected by finding the difference between the maximum points in a cycle of the original signal and the reconstructed one. After the compression algorithm was performed the best rates are 99.803% for compression rate, RTE 99.9479%, NMSE 0.000434, and Cross-Correlation 0.999925. Finally, this works presents two new performance criteria, compression time and recovery time, both of them in a real scenario will determinate how fast the algorithm can perform.

## 1 Introduction

Human migration from rural places to urban areas has been a constant phenomenon throughout human history. During the last decades, this phenomenon has evolved into migration from small cities to big ones. It is estimated that seven out of ten people will live in big cities over the next fifty years, this is by far the largest growth that metropolis has suffered. Commonly this is referred to as Urbanization and its implications for the environment, human quality life and energy consumption are a frequent topic for researchers around the world [3].

The fast pace of urbanization around the world is not entirely a good thing (millions of people overpopulating cities) because it might also drive to inequality resources distribution and lack of access to energy [3], [6]. To improve the quality of life for its inhabitants by managing efficiently its resources, cities are trying to figure it out the concept of “Smart Cities”. It is a widely accepted that a smart city final goal is to improve its inhabitant's quality life with the use of technology as a tool for eco-management of resources [3], [8].

By getting access to new technologies (such as new cellular networks, and new techniques for big data processing), smart cities can mitigate the impact of urbanization in the environment; both academia and industry centre their efforts on different areas, being the most important efficient energy management. Among the different parameters related to energy management, power quality is considered the most important and practical aspects in a smart city [5].

As a result of the massive growth of urbanization, a significant number of nonlinear loads are integrated into the power systems which reduce the predictability. For this reason, monitoring and analysis are the focus (regarding power quality) as they are necessary to detect and classify disturbances at any particular point of the power system [3], [5], [10].

Among the different reasons a disturbance might be caused, the most important ones could be attributed to systems electrical faults, capacitor-switching related events, switching events regarding non-linear loads, transformers inrush and natural disruptions. All these events translate into a poor system power quality that is perceived for the power system users in voltage sag, swell, harmonics, transients, voltage irruption, among others [5].

Any strategy to improve power quality begins with the power system being able to monitor all the electric variables (related to power quality) [7], [12]. For this purpose, it is necessary to deploy massive telecommunication systems such as: home area networks (HAN), Neighbourhood area networks (NAN), and Wide area network (WAN). Technological advantages have made it possible to have a massive number of sensors within these networks with a relatively low cost. However, a new challenge the communications networks are experienced is the capability to process, transport and store the enormous amount of data without losing any important information [4], [12].

Therefore, power quality control is possible, but to manage the massive amount of data the telecommunications networks are constantly acquiring it is necessary to study techniques for data compression which will save bandwidth (even with new technologies bandwidth is a limited resource) and also reduce costs extensively by avoiding to have massive data storage facilities [1], [5].

As previous works related to data compression techniques used for electrical signals in power quality management, the most important are listed as follows:

In [5], the author analyzes that flickers, harmonics, and transients provide non-stationary characteristics to the electrical power system, hence Fourier transform is not enough for non-stationary electrical signals analysis. This research uses a dual-tree complex wavelet transform (DTCWT). As a result, the compression ratio for voltage sag is 84%, 88% for voltage well, 82.87% for flickers, 68.75% for transients and 19.53 for harmonics.

In [9], the author proposes a data compression method related to wavelet decomposition and spline interpolation to process power quality disturbances. The technique consists of four stages: signal decomposition, thresholding of wavelet transform coefficients, the decimation of the last coefficient, and signal reconstruction using spline interpolation. As a result, the highest compression ratio of the signals is 63.99%.

In [16], the author proposes an improved regularization sparsity adaptive matching pursuit algorithm (RCoSaMP), this algorithm has a better performance when compared to other greedy algorithms (based on reconstruction speed and accuracy indexes). As a result, the highest compression ratio of the signals is 72%.

In [2], the author proposes improvements over the main steps that are usually implemented for automatic monitoring of disturbances in power quality. The results demonstrate the high performance of segmentation, classification and enhancements in power quality disturbance compression. As a result, the highest compression ratio of the signals is 25:1 with a performance of 56% better than traditional PQ compression techniques.

In [14] research, the author designs a method to restore lost signals under power failures events in transmission lines by using sensing techniques. The algorithm allows recovering the original signal from 70% of the random samples. Also, matching pursuit allows recovering the same percentage, but with a significant lower restoration time. Finally, a orthogonal matching pursuit method recovers a slightly lower percentage with a higher number of samples, and also increases the recovery time.

In [15], the author proposes two concepts as data compression techniques: a gapless power quality disturbance recorder (G-PQDR), and a novelty detector. The research works with signals of voltage sags, swells, even and odd harmonics. As the best result, the highest compression ratio of the signals is 570:1, without compression, the total size to store the signal would be 9.25 MB, however after the compression by the G-PQDR the size is 16.22 kB.

Henceforth, this article is organized as it follows. Section 2 presents the formulation of the problem. Section 3 presents the results. Section 4 analyzes the results of the model and its simulation. Finally, in section 5 presents research conclusions.

## 2 Problem formulation

Based on previous research works from different authors, there are plenty of time signal compression techniques that use the same orthogonal base as the signal in analysis to develop the data compression. Among the most common techniques for data compression found in the literature review, the most relevant are: Fourier transform, discrete cosine transform, wavelet transforms and disperse signals representation through compressed sensing [15].

Compression data rates do not depend on a unique parameter and they vary depending on the selected compression technique, specific signal characteristics (voltage, flickers, harmonics), sample frequency, etcetera. Among the results found in the literature review, compression for electrical signals in the time domain varies from 19.53% to 99.82% [2], [15].

Even though compression rates indicate how good the developed compression techniques work, it is also important to analyze the retained energy percentage (RTE) which is the relationship between the energy of the original signal (prior the compression) and the reconstructed signal, ideally, they should be as identical as possible. Among the results found in the literature review, RTE varies from 97.80% to 98.85% [2], [15].

Besides, the compression process must analyze the quality of the processed signals after the compression is done, the best and most common approach is to normalize the mean square error, therefore, a low NMSE corresponds to a small error between the reconstructed and the original one.

As for the time-series waveform itself, it is important to identify how similar is the reconstructed signal against the original one. Statistically, the cross-correlation is a measurement that tracks two variables relative to each other, therefore the cross-correlation in this paper will compare the pre-compressed signal and the reconstructed one, the range of this parameter varies from -1 to 1, the closer the value is to 1, the more close to each other the data sets are [11].

Thus, this research centres its focus on the improvement of compression data from electrical signals originated in a micro-grid. The compression algorithm centres its analysis in three parameters: RTE, NMSE and XCOR. To accomplish the research goal, this paper uses biorthogonal wavelet.

The biorthogonal wavelet is made of two processes, decomposition and reconstruction with two different wavelets *ψ* and $\overset{ˆ}{\psi }$. *ψ* is used for the decomposition and $\overset{ˆ}{\psi }$ is used for the reconstruction process, these two wavelets are dual and orthogonal with each other; this relationship is called biorthogonal. At the same time there are two scale functions *ϕ* and $\overset{ˆ}{\phi }$ related to the prior processes, these functions are also dual and orthogonal. In the same manner, one is used for decomposition and one for the reconstruction process. Therefore, by having two wavelets and two-scale functions, there are four filters in biorthogonal wavelet transform: decomposition low-pass filter $\left\{h_{n}\right\}$, decomposition high-pass filter $\left\{g_{n}\right\}$, reconstruction low-pass filter $\left\{\overset{˜}{h_{n}}\right\}$ and the reconstruction high-pass filter $\left\{\overset{˜}{g_{n}}\right\}$ [11], [13].

With these filter coefficients $\left\{h_{n}\right\}$, $\left\{g_{n}\right\}$, $\left\{\overset{˜}{h_{n}}\right\}$ and $\left\{\overset{˜}{g_{n}}\right\}$, fast wavelet transform can be performed. One wavelet, $\overset{ˆ}{\psi }$ is used in the analysis and the coefficients of a signal *s* are:

(1) $${\overset{˜}{c}}_{j,k}=\int s(x){\overset{˜}{\psi }}_{j,k}(x)dx$$

The other wavelet, *ψ* is used in the synthesis of the coefficients:

(2) $$s=\sum_{j,k}{\overset{˜}{c}}_{j,k}⁎\psi_{j,k}$$

Additionally, the two wavelets are related by duality in the following sense:

(3) $$\int {\overset{˜}{\psi }}_{j,k}(x)⁎\psi_{j^{\prime },k^{\prime }}(x)dx=0$$

As soon as $j\neq j^{\prime }$ or $k\neq k^{\prime }$ and:

(4) $$\int {\overset{˜}{\phi }}_{0,k}(x)⁎\phi_{0,k^{\prime }}(x)dx=0$$

As soon as $k\neq k^{\prime }$.

In the present investigation, the moving average shown in equation (5) was applied to eliminate the ripple. The value of N varies according to the wavelet levels used. For example, the value of N at level 1 is 33 while the value of N at level 6 is 66.

(5) $$MoveMean\;=\;\frac{1}{N}\sum_{i=1}^{N}reconstructed\_signal_{i}$$

To evaluate the quality of the reconstructed signal the following quantities are used:

Equation (6) shows the percentage of retained energy (RTE), the reconstructed signal is more similar to the original one if the RTE is close to 1, which is also represented as 100%.

(6) $$RTE(\text{\%})\;=\;\frac{\sum_{n=0}^{N}x{\left[n\right]}^{2}}{\sum_{n=0}^{N}\overset{ˆ}{x}{\left[n\right]}^{2}}$$

or Equation (7) shows the percentage of recovery between two signals.

(7) $$Energy\;Recovery(\text{\%})\;=\;\frac{100\cdot ||\overset{ˆ}{x}||}{\left||x|\right|}$$

Equation (8) shows the Normalized Mean Square Error (NMSE), the algorithm performance is better if the reconstructed signal has a NMSE close to 0.

(8) $$NMSE\;=\;\frac{||x-\overset{ˆ}{x}|{|}^{2}}{||x|{|}^{2}}$$

Equation (9) shows the cross-correlation (XCOR) between the reconstructed signal and the original signal, the reconstructed signal is more similar to the original one if the XCOR is close to 1, which is also represented as 100%.

(9) $$XCOR\;=\;\frac{x^{T}\cdot \overset{ˆ}{x}}{x^{T}\cdot x}$$

Algorithm 1 shows the initial process necessary for every electrical signal. First, data is acquired and stored in OS “Original Signal”. Then, the number of samples per signal and the number of signals are calculated and these values are stored in ROS “Row Original Signal” and COS “Column Original Signal” respectively. The next step consists in finding the signal indexes, these values correspond to the zero-crossing and they are stored in A and B. The indexes represent an entire cycle of the original signal (before compression), they allow calculating the maximum peak value of a cycle and its position; these indexes are stored in C and D. Finally, the number of samples per cycle is calculated and it is stored in the E index. It is important to emphasize that this process must be carried out for each phase of the electrical signals since they are 120 degrees out of phase between each other.

**Algorithm 1:**
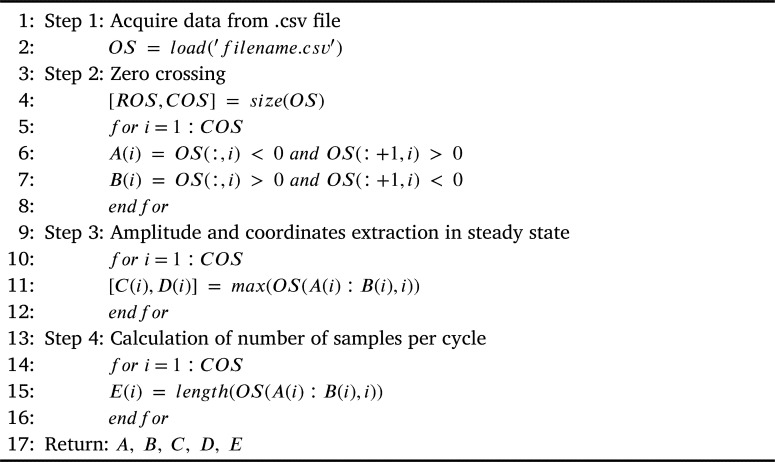
Signal characteristics extraction.

**Figure 1:**
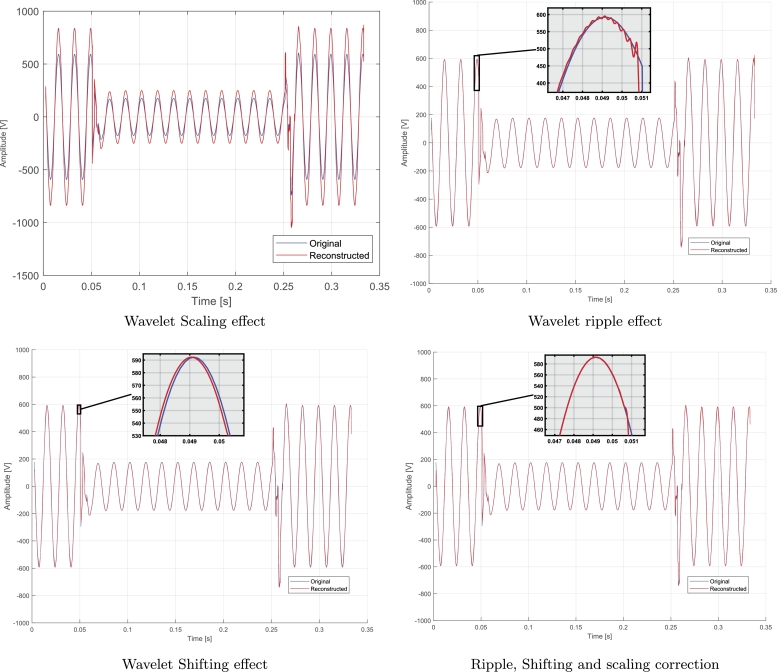
Wavelet Faults.

Algorithm 2, shows the steps necessary for signal compression by using wavelets. The first step consists in taking one by one the original electrical signals and applying them a wavelet bior1 level 6 for compression. The result is a compressed signal but as an effect of the wavelet, the compressed signal has a different amplitude from the original one, also ripple and shifting are added when compared with the original signal. To correct the amplitude of the compressed signal, this signal is normalized by using the maximum peak value of the first cycle previously calculated in Algorithm 1, and then it is divided by the maximum peak value of the first cycle of the compressed signal. Then, this result is multiplied by the compressed signal and the result is stored in SCN “Signal Compressed Normalized”, this is shown in Fig. 1 literal b. To eliminate the ripple, a moving average filter has been used; for the process, it is necessary to calculate the number of samples taken by the filter and this value is stored in N, in this paper N, is equal to 0.1% of the size of the compressed signal. Then, the result is stored in SCNA “Compressed Normalized Average Signal” and it is shown in Fig. 1 literal c. Finally, the shifting is corrected by calculating the difference in time between the indexes of the maximum peak values of the original signal and the compressed one. The result is stored in the SCNAS matrix “Compressed Normalized Average Shifting Signal” and is shown in Fig. 1 literal d.

**Algorithm 2:**
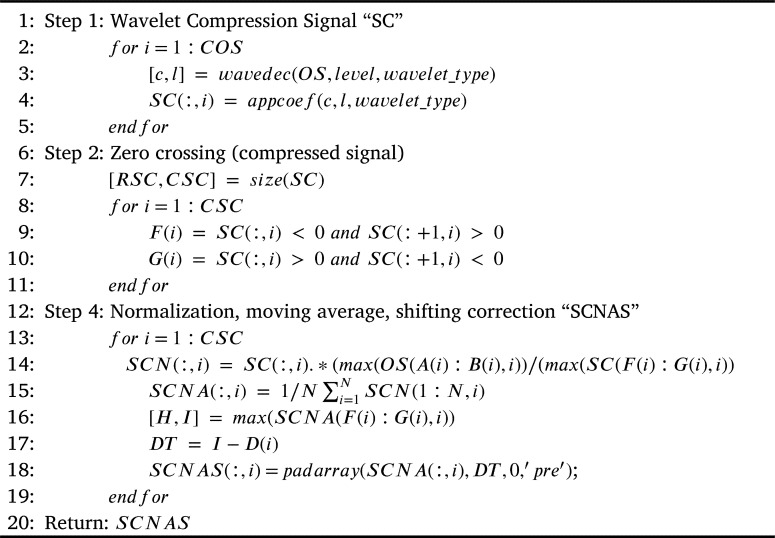
Wavelet compression of an electrical signal.

Algorithm 3 presents the compression of the signals by using the windowing. The first step is to calculate the signals samples size, J and K are the number of samples per signal and the number of signals respectively. In Algorithm 3, OSP represents one cycle of the original signal, SCP represents one cycle of the wavelet compressed signal. Then, the frequency of the original signal is compared one by one with all cycles of the compressed signal. If RTE is high, between 0.9999 and 1.0009 when comparing the two signals, it indicates that both signals are identical and then zeros are placed in that signal cycle in the SCW matrix, by doing this process a vector is created, this vector represents an index that will later allow the reconstruction. On the contrary, if RTE is below 0.9999, the values of the signal are placed for that cycle in the SCW matrix. By doing this process, equal signals (high RTE), are eliminated, allowing further compression of the signals.

**Algorithm 3:**
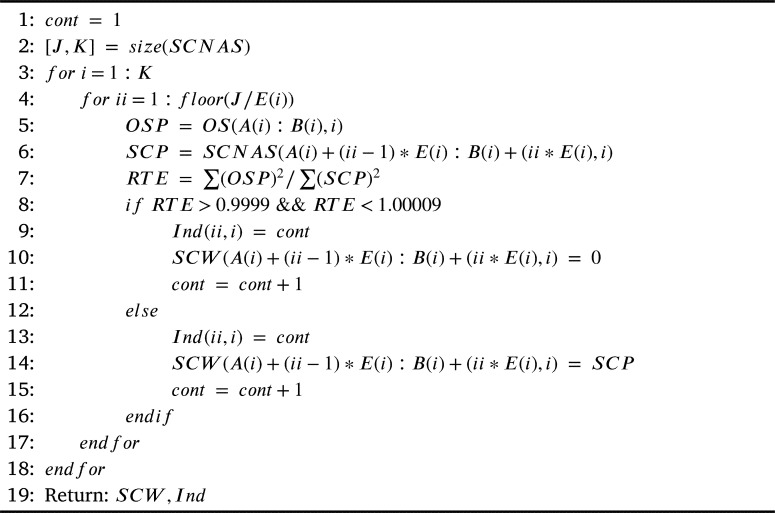
Windowing for elimination of repeated signals.

Algorithm 4 presents the reconstruction of the signal, the first step is to calculate the number of signals and the number of samples per signal, these data are stored in the variables L and M. A for loop is made from 1 to M, allowing reconstruction of all signals. The second FOR loop from 1 to E (i) indicates the actual size of each signal and how far the signals should be reconstructed. With the third FOR loop from 1 to the maximum number of indexes per signal, the indexes represent the number of repeating periodic signals and the order of repetition. The variable “ni” stores the value of the index. The variable SC stores the cycles of the SCW signal. If the value of variable “iii” is equal to index “ni”, the signal cycle stored in SC is placed. In other words, each cycle of the SCW signal is reviewed and the original signal is reconstructed based on the indexes. Finally, the RTE, Energy Recovery, NMSE and XCOR are calculated to verify the relationship indexes between the original signal and the reconstructed signal.

**Algorithm 4:**
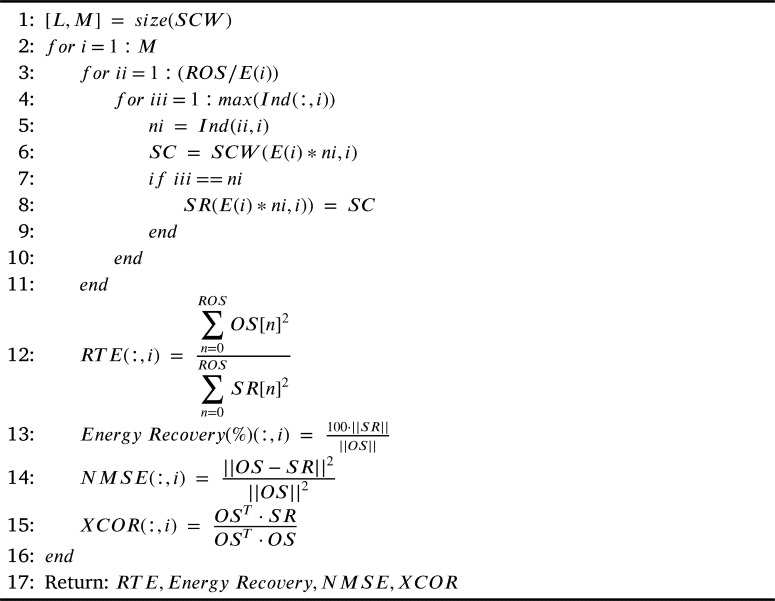
Reconstruction.

From the algorithm described 1, two values are obtained: steady-state maximum magnitude value and position for both the original and the compressed signal. Finally, the reconstructed signal is multiplied by the result of the division between the original signal maximum magnitude and the reconstructed signal maximum magnitude as it is shown in Fig. 1 literal a.

The next problem to be solved is the ripple that exists in the reconstructed signal as it is shown in Fig. 1 literal b, when zooming in on the maximum magnitude points of each electrical phase. To solve this problem, it is proposed to apply the moving average filter described in the (5) or apply the moving average calculation.

Finally, due to the characteristics of the wavelet transformation, specifically, change of scale and translation compared to the original signal, a slight shifting between the original signal and the reconstructed signal can be evidenced when zooming in, as it can be seen in Fig. 1 literal c. To correct the shifting presented between the signals, the approach this paper proposes is to use the positions of the maximum points calculated in a cycle of the signal in the original steady-state and the reconstructed one. The difference between the positions of these two points allows the reconstructed signal to be shifted until they stay in phase, as can be seen in Fig. 1 literal d.

## 3 Results

To analyze variables related to power quality, this research proposes a 200 kHz sampling rate, this allows the measurement of very fast signals such as transients. Transients are produced by atmospheric discharges, they are presented as pulses with duration in the order of microseconds, and they typically can last from 50 ns to 1 ms. Besides, by using the same sampling rate, three more signals have been analyzed: voltage signals in steady-state, a three-phase fault and swell.

**Figure 2:**
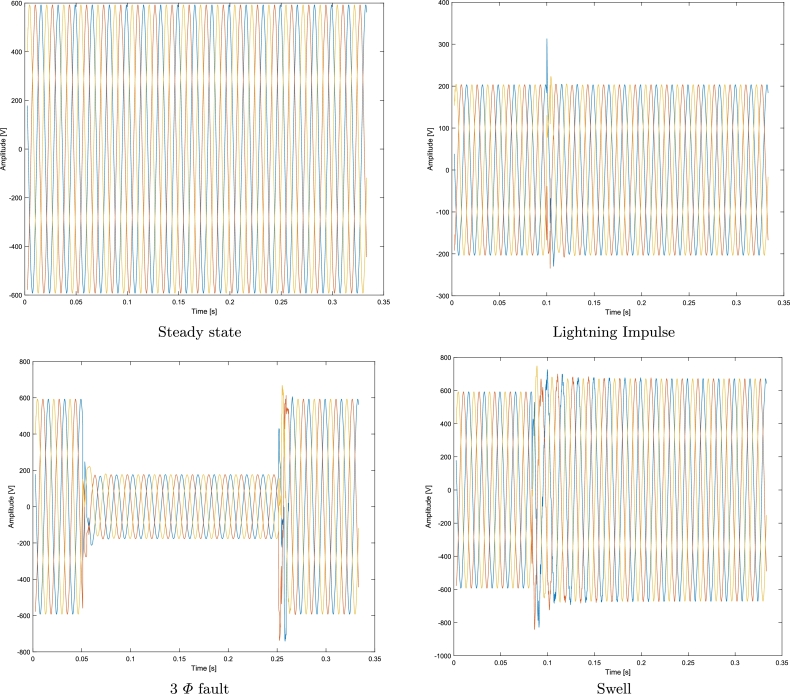
Original signals.

Each signal analyzed generates a 66168x4 matrix, every column carries specific information regarding with time, voltage R, voltage S and voltage T respectively. Furthermore, the files generated have a size of 1,889,931 bytes. Finally, the computer equipment used in the experiment development has the following characteristics: Processor (Intel (R) Xeon (R) E-2176M CPU @ 2.70GHz), and 64 GB RAM. Fig. 2 shows the representation of each signal as a function of time.

**Figure 3:**
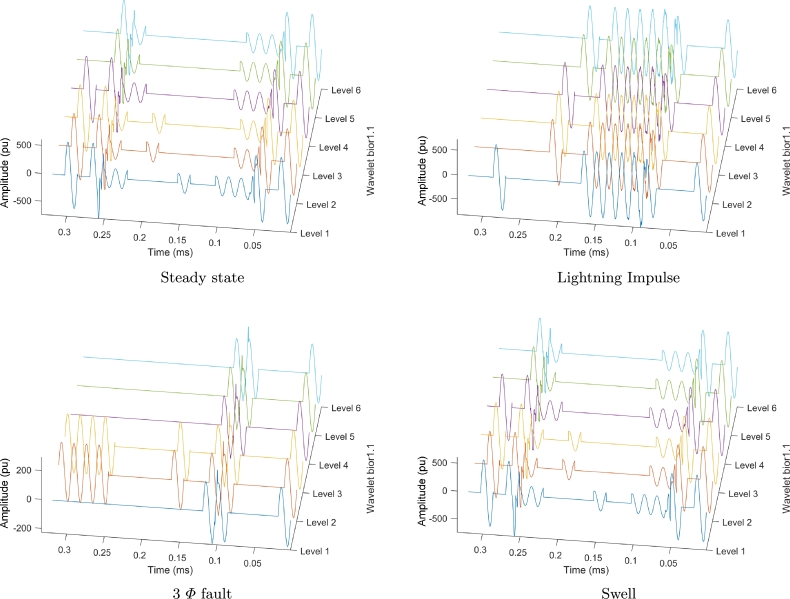
Different wavelet levels for reconstruction.

Fig. 3 literal a, shows the compressed signal in steady-state by using wavelet bior1.1, it can be seen that in steady-state, it is necessary to have the information from the first cycle and depending on the wavelet level, the algorithm selects the necessary signals for the subsequent reconstruction.

Fig. 3 literal b shows several signals that are necessary to reconstruct a disturbance caused by the phenomenon of atmospheric discharge. It can be seen that the number of signals per cycle increases compared to a single signal in steady-state; it is also shown that there are cycles in which the signals are zero because they are represented by another signal while maintaining a high level of RTE.

Fig. 3 literal c, represents the signal of a three-phase fault, it is observed that the fault generates a phase shift when compared to the steady-state signal, this makes necessary a large number of signals represented by cycles to reconstruct the original signal.

Fig. 3 literal d, shows the result of the compression algorithm and the proposed windowing for disturbances such as sag, swell or harmonics, this solution is proposed by considering that after a certain time the system tends to stabilize itself and future signals can be represented by specific signals in certain cycles.

Finally, in all figures (a, b, c and d), it can be observed that as the wavelet level increases, fewer sample cycles are necessary because some characteristics of small amplitude signals are lost, allowing just a few signals (being necessary) to represent the whole set.

**Figure 4:**
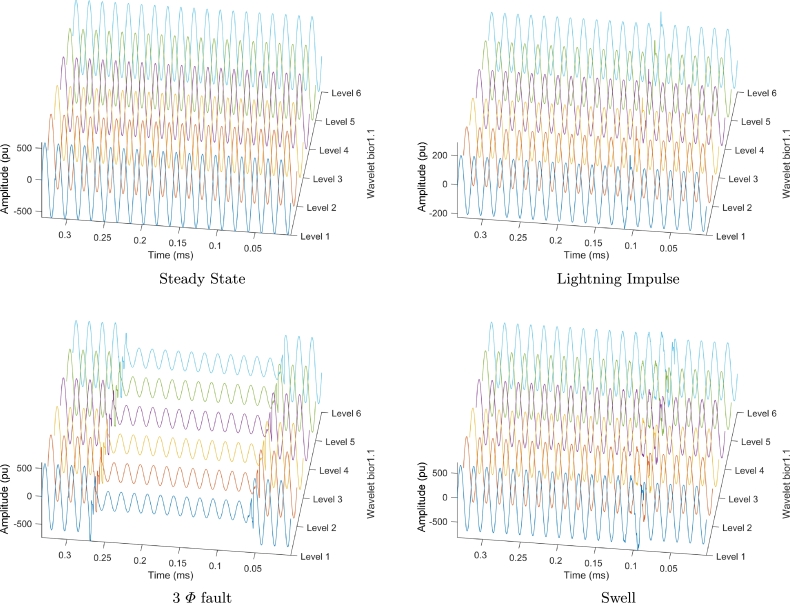
Signals reconstruction.

Fig. 4 shows the reconstruction of each of the analyzed signals represented in the six levels of the wavelets. Literal a shows the compressed signal in steady-state, literal b shows the compressed signal in lightning impulse, literal c shows the compressed signal in 3 Φ fault, literal d shows the compressed signal in swell. It can be noted that there is a similarity between the different levels of each of the signals, thus, corroborating the results shown in Tables 1 and 2.

**Table 1:** Comparison of Compression results between different Wavelet levels.

Wavelet Compression in bytes						
Signal	Level 1	Level 2	Level 3	Level 4	Level 5	Level 6

All signals	972,522	483,306	250,021	118,528	61,602	30,670
Compression %	48.5419	74.4273	86.7790	93.7284	96.740	98.377

Windowing in bytes						

Steady State	41.547	21,504	30,624	16,035	8,793	3,723
Compression %	97.8017	98.8622	98.379	99.151	99.534	99.803

Lightning	117,321	59,706	39,954	30,579	20,559	4,959
Compression %	93.7923	96.8408	97.886	98.382	98.912	99.737

Fault 3Φ	269,043	135,867	87,885	40,089	20,757	13,329
Compression %	85.7644	92.8110	95.349	97.878	98.901	99.294

Swell	306,348	154,674	78,447	39,762	20,625	10,929
Compression %	83.7905	91.8159	95.849	97.896	98.908	99.421

**Table 2:** Compression results: RTE, NMSE, Cross correlation Compression Time and Recovery Time.

Bior 1.1	RTE (%)	NMSE	Cross Correlation	Compression Time	Recovery Time
Steady State Signal					

Level 1	99.9143	0.019565	0.990646	0.259778	0.290082
Level 2	99.9059	0.001650	0.999645	0.092780	0.443042
Level 3	99.9357	0.001768	0.999437	0.082618	0.134150
Level 4	99.9315	0.001972	0.999356	0.048862	0.049667
Level 5	99.9486	0.002044	0.998720	0.039826	0.086259
Level 6	99.9479	0.001902	0.998788	0.036617	0.101389

Lightning Signal					

Level 1	99.8847	0.011983	0.994585	0.283787	0.445981
Level 2	99.8620	0.001972	0.999704	0.130686	0.482466
Level 3	99.8861	0.002771	0.999183	0.042138	0.089791
Level 4	99.8814	0.000380	0.999216	0.039053	0.114254
Level 5	99.9988	0.000098	0.999944	0.039361	0.231550
Level 6	99.9415	0.000434	0.999925	0.048162	0.115147

Fault 3Φ Signal					

Level 1	99.77542	0.013614	0.992070	0.244907	0.246346
Level 2	99.90412	0.006783	0.996128	0.120452	0.445679
Level 3	99.93682	0.008608	0.995380	0.039305	0.110532
Level 4	99.95180	0.007468	0.996024	0.054989	0.091779
Level 5	99.84289	0.003184	0.997622	0.074945	0.097257
Level 6	99.84292	0.002965	0.997731	0.052374	0.113188

Swell Signal					

Level 1	99.9383	0.004697	0.997960	0.521757	0.794249
Level 2	99.9103	0.003014	0.998941	0.089559	0.409472
Level 3	99.9314	0.004772	0.997957	0.050329	0.126874
Level 4	99.9210	0.003857	0.998466	0.116466	0.065097
Level 5	99.9595	0.000132	0.999731	0.077322	0.157097
Level 6	99.9825	0.001105	0.999534	0.036522	0.144762

Table 1 shows the compression performed by the wavelet at each level. From the data shown in Table 1, it is evident that the compression percentage for each signal is the same at every level, furthermore, this percentage does not depend on the characteristics of the signal to be analyzed. In the windowing section, the size of each signal is presented in bytes and it can be seen that the compression level does not only depend on the wavelet level but also on the characteristics of each of the signals to be analyzed.

Table 2 provides a summary of the results obtained from the signals analyzed: steady-state, atmospheric discharge, three-phase faults and swell. The results of RTE, NMSE, and Cross-correlation are shown for each wavelet level (these four parameters are found in the literature review). In addition, this research considers it important to analyze two additional parameters that have not been considered in previous works. These parameters are: “Compression Time” which is the time taken from the moment the file (with the original data) is loaded until the generation of a new file with the compressed signal; and, “Recovery Time” which is the time from the moment the file with the compressed signal is loaded until the generation of a new file with all the information of the reconstructed signal.

## 4 Analysis of results

The Q1 quartile high impact investigation [15], has been taken as a reference goal to be achieved and surpassed, this is because, in the literature review, the mentioned work has the best results so far (considering data compression) considering SCOPUS, ELSEVIER and SPRINGER databases. A summary of the article is shown in Table 3, these values will help to contrast the results obtained in the present investigation.

**Table 3:** Previous work from literature review: Gapless Power-Quality Disturbance Recorder [15].

Summary: Q1 paper				
Signal	RTE (%)	NMSE	Cross Correlation	Compression

Steady State	97.80	0.0277	0.989	99.776
Sags, Swells	98.37	0.0343	0.991	99.159
harmonics	98.54	0.0270	0.992	99.824
frequency variation	98.85	0.0700	0.992	97.872

The Q3 quartile investigation [2] is another work that has been taken as a reference goal to be achieved and surpassed. A summary of the article results is shown in Table 4, to contrast with the values obtained in the present investigation.

**Table 4:** Previous work from literature review: Advances in Classification and Compression of power quality Signals [2].

Summary: Q3 Paper				
Signal	RTE (%)	NMSE	Cross Correlation	Compression

Steady state	Not specified	1.0 ×10^−5^	Not specified	94.117
Sags, Swells	Not specified	1.0 ×10^−5^	Not specified	97.297
harmonics	Not specified	1.0 ×10^−5^	Not specified	96.296
frequency variation	Not specified	1.0 ×10^−5^	Not specified	97.297

Also, in Table 5 a summary is presented with the best results of the experiment carried out in the present investigation.

**Table 5:** Most important results obtained in the present paper.

Present work Summary				
Signal	RTE (%)	NMSE	Cross Correlation	Compression

Steady state	99.9479	0.001902	0.998788	99.803
Lightning	99.9415	0.000434	0.999925	99.737
Fault 3Φ	99.84292	0.002965	0.997731	99.294
Sags, Swells	99.9825	0.001105	0.999534	99.421

The results that are shown in Table 5, evidence that the compression level exceeds 99.294% in all the proposed scenarios. These results excel previous works form the papers, Q1 and Q3 in three cases (Tables 3 and 4). Only the harmonic compression in article Q1 (Table 3) exceeds the results of the present investigation.

The RTE obtained in the present investigation exceeds 99.84% in all the scenarios carried out, so it can be concluded that there is a better reconstruction of the signal compared to article Q1, which presents 98.85% as its best result

The cross-correlation in the present investigation exceeds 0.992, which is precisely the best value of the contrasted article (Q1).

The highest NMSE in the present investigation is 0.00296, improving the values of the contrasted article that at its best presents 0.0270 (Q1 article).

Although the NMSE of the article “Advances in Classification and Compression of Power Quality Signals” is lower when compared to this research results; in this work, the compression levels are obtained by using the wavelet transform, therefore the compression levels of this are higher.

## 5 Conclusions and future works

Electrical power signal compression through the proposed algorithm allows obtaining compression ratios of 99.803%.

In the literature review, most of the authors concluded at first glance, that the reconstructed signal is similar to the original one, however, they have not taken into account aspects such as the change in amplitude, the ripple generated or the shift in the signal, all caused by characteristics of wavelets, this research eliminates those effects as it is described in Algorithm 2.

The algorithms proposed in this research (before performing the windowing) have made it possible to improve the quality indices of the reconstructed signal such as RTE, NMSE, and X-COR when compared to previous results cited in the literature review [15].

Finally, the proposed windowing in which signals that are repeated by cycles are searched has allowed getting higher compression ratios and by doing this, improving the indexes proposed in the literature review.

For future works, it is proposed to apply compressed sensing techniques to show if there is the possibility of further compressing the signal (by getting higher compression ratios) while maintaining or improving the RTE, NMSE COR parameters.

**Nomenclature**

The Table 6 presents a summary of the power flow analysis, including the power source, the load installed in each zone, the conductor ampacity, and the losses in lines, conductors and transformers.

**Table 6:** Used variables.

Matrix	
OS	Original Signal
SC	Compressed Signal
SCN	Compressed normalized signal(ripple eliminated)
SCND	Compressed normalized signal(ripple eliminated and shifting correction)
SVC	Compressed signal during windowing
SR	Reconstructed signal

Variables	

A	Original signal beginning index cycle
B	Original signal ending index cycle
C	Original signal maximum value (in a cycle)
D	Original signal maximum value index (in a cycle)
E	Number of samples per cycle
c	Wavelet decomposition vector
l	Bookkeeping vector
F	Compressed signal beginning index cycle
H	Compressed single ending index cycle
I	Compressed signal maximum value (in a cycle)
J	Compressed signal maximum value index (in a cycle)
NC	Number of cycles of the compressed signal
ni	Index of the number of cycles
sni	Indexes length

## Declarations

### Author contribution statement

Milton Ruiz: Conceived and designed the experiments; Performed the experiments. Silvio Simani & Esteban Inga: Analyzed and interpreted the data. Manuel Jaramillo: Contributed reagents, materials, analysis tools or data; Wrote the paper.

### Funding statement

This work was supported by Universidad Politécnica Salesiana.

### Data availability statement

Data included in article/supp.material/ referenced in article.

### Declaration of interests statement

The authors declare no conflict of interest.

### Additional information

No additional information is available for this paper.
